# Vascular normalization in orthotopic glioblastoma following intravenous treatment with lipid-based nanoparticulate formulations of irinotecan (Irinophore C™), doxorubicin (Caelyx^®^) or vincristine

**DOI:** 10.1186/1471-2407-11-124

**Published:** 2011-04-08

**Authors:** Maite Verreault, Dita Strutt, Dana Masin, Malathi Anantha, Andrew Yung, Piotr Kozlowski, Dawn Waterhouse, Marcel B Bally, Donald T Yapp

**Affiliations:** 1 Experimental Therapeutics, British Columbia Cancer Agency, 675 West 10thAvenue, Vancouver, BC V5Z 1L3, Canada; 2 Faculty of Pharmaceutical Sciences, University of British Columbia, 2146 East Mall, Vancouver, BC V6T 1Z3, Canada; 3 Department of Pathology and Laboratory Medicine, University of British Columbia, 2211 Wesbrook Mall, Vancouver, BC V6T 2B5, Canada; 4 Center for Drug Research and Development, Vancouver, BC V6T 1Z4, Canada; 5 UBC MRI Research Center, 2221 Wesbrook Mall, Vancouver, BC V6T 2B5, Canada

**Keywords:** glioblastoma multiforme, vasculature normalization, liposomal drugs, endothelial cells

## Abstract

**Background:**

Chemotherapy for glioblastoma (GBM) patients is compromised in part by poor perfusion in the tumor. The present study evaluates how treatment with liposomal formulation of irinotecan (Irinophore C™), and other liposomal anticancer drugs, influence the tumor vasculature of GBM models grown either orthotopically or subcutaneously.

**Methods:**

Liposomal vincristine (2 mg/kg), doxorubicin (Caelyx^®^; 15 mg/kg) and irinotecan (Irinophore C™; 25 mg/kg) were injected intravenously (i.v.; once weekly for 3 weeks) in Rag2M mice bearing U251MG tumors. Tumor blood vessel function was assessed using the marker Hoechst 33342 and by magnetic resonance imaging-measured changes in vascular permeability/flow (K_trans_). Changes in CD31 staining density, basement membrane integrity, pericyte coverage, blood vessel diameter were also assessed.

**Results:**

The three liposomal drugs inhibited tumor growth significantly compared to untreated control (p < 0.05-0.001). The effects on the tumor vasculature were determined 7 days following the last drug dose. There was a 2-3 fold increase in the delivery of Hoechst 33342 observed in subcutaneous tumors (p < 0.001). In contrast there was a 5-10 fold lower level of Hoechst 33342 delivery in the orthotopic model (p < 0.01), with the greatest effect observed following treatment with Irinophore C. Following treatment with Irinophore C, there was a significant reduction in K_trans _in the orthotopic tumors (p < 0.05).

**Conclusion:**

The results are consistent with a partial restoration of the blood-brain barrier following treatment. Further, treatment with the selected liposomal drugs gave rise to blood vessels that were morphologically more mature and a vascular network that was more evenly distributed. Taken together the results suggest that treatment can lead to normalization of GBM blood vessel the structure and function. An *in vitro *assay designed to assess the effects of extended drug exposure on endothelial cells showed that selective cytotoxic activity against proliferating endothelial cells could explain the effects of liposomal formulations on the angiogenic tumor vasculature.

## Background

Glioblastoma (GBM) tumors are largely refractory to systemic treatments; the median survival time for patients with GBM is 10 months and the 2-year survival rate is less than 10%. Chemotherapy for GBM is compromised in part by the blood-brain barrier limiting drug access to the malignant cells. In addition, pre-clinical models showed that GBM tumors are poorly perfused [1,2] due to factors such as reduced blood flow rates, elevated hematocrit and interstitial fluid pressure, and an increase in geometric resistance [3-6], all of which impede drug delivery to the tumor tissue. Strategies which improve vascular function in GBM tumors should improve the delivery of other drugs capable of crossing the blood brain barrier and this should be associated with an increase in therapeutic activity.

Our laboratory has previously characterized and described the effects of a liposomal formulation of irinotecan (Irinophore C™) [7,8]. Encapsulation of irinotecan into liposomes improved the pharmacokinetic profile of the drug and its active metabolite, SN-38. More specifically, administration of Irinophore C™ resulted in a 1000-fold increase in the area-under-the-curve of plasma irinotecan concentration when compared to free drug (Camptosar). In addition, following irinophore C™ injection, the plasma levels of SN-38 were maintained at concentrations that were up to 40-fold higher than that achieved following injection of free drug [7]. Following irinophore C™ treatment, the s.c. (subcutaneous) colorectal tumors (HT-29) exhibited more functional tumor blood vessels, reduced hypoxia, and increased tumor perfusion. Importantly, these changes in tumor vasculature were associated with increased tumor uptake of doxorubicin and 5-FU given intravenously [8]. The latter data were consistent with the idea that the tumor vasculature in the treated tumors acquires a more "normal-like" function; an effect of anti-angiogenic therapies described as 'normalization' [9,10].

The primary goal of the studies reported here was to determine whether Irinophore C™ is efficacious in models of GBM, and whether treatment with this drug formulation would also result in normalization of GBM vasculature. The effects of Irinophore C™ on the growth rates and vascular function of the HT-29 colorectal cancer model was attributed to significant increases in the drug circulation lifetime and plasma concentration when encapsulated in liposomes [7,8]. We further reasoned that liposomal formulations of other drugs with known activity against proliferating endothelial cells should have preferential cytotoxicity towards angiogenic tumor vessels and could potentially also 'normalize' the chaotic and erratic vasculature of tumors. Thus, part of these studies assessed the effects of liposomal vincristine [11] and doxorubicin (Caelyx^®^) on tumor vasculature. Vincristine has previously been shown to be active against proliferating endothelial cells [12]. Liposomal formulations of doxorubicin have also been shown to have direct effects on tumor associated vasculature [13-15].

The data reported here assess the effects of Irinophore C™, Caelyx^® ^(a commercially available and FDA-approved liposomal formulation of doxorubicin), and liposomal vincristine on tumor vasculature in subcutaneous and orthotopic models of GBM. The results indicate that Irinophore C™ was the most active formulation when using treatment endpoints based on changes in tumor size as well as tumor vascular morphology and function in GBM grown subcutaneously and orthotopically. The effects were consistent with the idea that following treatment, there was normalization of tumor vasculature. In the subcutaneous tumors, vascular 'normalization' was associated with increased tumor uptake of Hoechst 33342, while in the orthotopic glioma tumors, treatment-induced vascular 'normalization' was associated with decreased tumor uptake of Hoechst 33342.

## Methods

### Cell culture

Adult dermal human microvascular endothelial cells (d-HMVEC; Cambrex Bio Science, Walkersville, MD), Human brain microvascular endothelial cells (HBMEC; ScienCell Research Laboratories, San Diego, California) and U251MG glioblastoma cells (American Type Culture Collection, Manassas, VA) were characterized and authenticated by the cell banks using immunofluorescent methods and used for a maximum of eight passages for the endothelial cells and fifteen passages for U251MG. Stock cells lines were maintained in the absence of penicillin and streptomycin and screened for mycoplasma prior to preparing a stock of cells that were frozen for use in experiments. D-HMVEC cells were maintained in Endothelial Cell Basal Medium-2 (Clonetics^®^, Lonza, Basel, Switzerland) supplemented with 5 ng/mL Fibroblast Growth Factor, 20 ng/mL Vascular Endothelial Growth Factor, 10 ng/mL Epidermal Growth Factor (Clonetics^®^, Lonza), 10 unit/mL Heparin (Pharmaceutical Partners of Canada) 1% L-glutamine, 1% penicillin/streptomycin (Stem Cell Technologies, Vancouver, BC, Canada) and 10% Fetal Bovine Serum (FBS; Hyclone, Logan, UT), and plated in 1% gelatin (Sigma, Oakville, ON, Canada) pre-coated dish. HBMEC cells were maintained in Endothelial Cell Medium supplemented with Endothelial Cell Growth Supplement (ScienCell Research Laboratories) containing 5 μg/mL Insulin, 10 ng/mL Epidermal Growth Factor, 2 ng/mL Fibroblast Growth Factor, 2 ng/mL Insulin-like Growth Factor-1, 2 ng/mL Vascular Endothelial Growth Factor, 1 μg/mL hydrocortisone, 5% FBS and 1% penicillin/streptomycin, and plated in 15 μg/mL fibronectin (Sigma) pre-coated dish. U251MG cells were maintained in DMEM medium supplemented with 1% L-glutamine, 1% penicillin/streptomycin (Stem Cell Technologies, Vancouver, BC, Canada) and 10% FBS (Hyclone, Logan, UT). All cell lines were cultured at 37°C in a humidified atmosphere containing 5% CO_2_, and used during exponential growth phase unless otherwise stated.

### GBM animal model s.c. and orthotopic

All protocols involving work with live animals were reviewed and approved by the University of British Columbia Animal Care Committee (certificate of approval # A07-0423). For the subcutaneous GBM model, U251MG cells (5 × 10^6^) were implanted subcutaneously into the backs of Rag2M mice (7-10 weeks old females, n = 9). To generate orthotopic GBM tumors, U251MG (7.5 × 10^4^) cells were implanted into the right caudate nucleus-putamen (ML -1.5 mm; AP +1 mm; DV -3.5 mm) of mice (n = 5-6) using a stereotaxic injection frame (Stoelting Company, Wood Dale, IL). Animals were treated with 25 mg/kg Irinophore C™, 2 mg/kg liposomal vincristine or 15 mg/kg doxorubicin liposome (Caelyx^®^, Schering-Plough, QC, Canada) i.v. on day 21, 28 and 35 after inoculation. Dosing of liposomal vincristine and Caelyx^® ^resulted in less than 5% body weight loss, while Irinophore C™ treatment did not cause any change in body weight. Previous tests in our laboratory have shown that the maximum tolerated single doses for Irinophore C™, Caelyx^® ^and liposomal vincristine are >120 mg/kg, 17 mg/kg and 3 mg/kg, respectively. Irinophore C™ [16] and liposomal vincristine [17] were prepared as described previously. S.c. tumor size was measured throughout the study by caliper and tumor weights were extrapolated from the measurements using the following formula: mg = (tumor width^2 × tumor length)/2 [18]. Mice were injected with Hoechst 33342 (1.2 mg/mouse; Sigma) twelve (s.c. model) or twenty (orthotopic model) minutes prior to sacrifice on day 42. This timing was chosen based on previous study [8] and tests (not shown) aimed at determining the optimal timing for Hoechst 33342 injection without saturation of the tissue and before any decrease in Hoechst 33342 staining could be observed due to possible metabolic elimination. All animals were terminated by CO_2 _asphyxiation and s.c. tumors or brains were harvested and cryopreserved in OCT (Sakura Finetek, CA) on dry ice and stored at -80°C.

### Hoechst 33342, Ki67, CD31, VEGFR2, EF5, Collagen IV, NG2 and nuclei density staining and quantification

Optimal Cutting Temperature compound (OCT)-preserved s.c. tumors were cryosectioned using a Leica CM1850 Cryostat (Leica, ON, Canada) and 10 μm sections were collected in the middle of each tumor. OCT preserved brains were cryosectioned and 10 μm sections were collected from the Bregma +1.0 location. Sections were fixed in a 1:1 mixture of acetone:methanol for 15 minutes at room temperature, then blocked with blocking buffer (Odyssey blocking buffer, Rockland, PA) for 1 hour at room temperature. Sections were stained with rat anti-mouse CD31 antibody (1:100 dilution, PharMingen #550274, BD Biosciences), rabbit anti-human Ki-67 (Invitrogen #18-0191z; 1:100), rabbit anti-human/mouse vascular endothelial growth factor receptor 2 antibody (VEGFR2; 1:100; Cell Signaling technology #2479, NEB, Pickering, ON, Canada), rabbit anti-Collagen IV antibody (1:400, Abcam # ab19808, Cambridge, MA) and mouse anti-NG2 chondroitin sulfate proteoglycan antibody (1:100, Millipore # MAB5384, Billerica, MA). Primary antibodies were incubated on sections overnight at 4°C. Secondary antibodies (Alexa 488 goat anti-rat #A11006, Alexa 546 goat anti-rabbit #A-11035 and Alexa 633 goat anti-mouse #A-21126, 1:200, Invitrogen) were incubated for 1 hr at room temperature. Nuclei were stained with Draq5 (Biostatus, Leicestershire, UK; 1:200) for 30 min at 37°C. Slides were mounted with PBS and imaged for Alexa 488 (L5 filter), Hoechst 33342 (A4 filter), Alexa 546 (Cy3 filter), Cy5 (Cy5 filter) and Draq5 (Cy5 filter) using a robotic fluorescence microscope (Leica DM6000B, Leica, ON, Canada) and a composite color image of these markers was produced (Surveyor software, Objective Imaging Ltd.). Thresholds for each marker were set using Photoshop; the threshold level was set using a scale from 1 to 255 units, and was defined at 2 units higher than the minimal level necessary to obtain a negative signal for non-specific staining, and was kept the same for all sections. Acquired images were quantified for positive pixels or colocalization (double-positive pixels) using an in-house segmentation algorithm, normalized to the number of pixels in the tumor area and expressed as positive fraction (positive pixels divided by non-necrotic tumor area; MATLAB, The Mathworks, Natick, MA). Non-necrotic tumor areas were defined by cropping out necrotic and non-tumor tissue on the basis of positive Ki-67 and Draq5 co-stained sections and were quantified using the same in-house algorithm. Colocalization was considered positive when two positive pixels from one stain of interest were located within a 3 pixels radius from one pixel of the other stain of interest. Of note, one cell nucleus measures between 3 and 6 pixels. Blood vessel diameter was defined by taking 10 measurements/tumor section in a 15 × 15 cm box at 200% magnification using Photoshop, and was expressed in pixels. For differential analysis between the tumor's center and periphery, the boundary between the tumor center and periphery area was established at 20% of tumor diameter distance from tumor margin. Another set of sections was stained with hematoxylin and eosin for histopathology analysis. The fraction of collagen IV-free blood vessels was defined as Collagen IV negative/CD31 positive pixels over total CD31 pixels. The fraction of NG2-free blood vessels was defined as NG2 negative/CD31 positive pixels over total CD31 pixels. The amount of basement membrane empty sleeves was defined as CD31 negative pixels/collagen IV positive pixels divided by the total non-necrotic tumor area.

### Magnetic Resonance Imaging and K_trans _measurement in U251MG orthotopic tumors

All magnetic resonance experiments were carried out using a 7.0 Tesla MR scanner (Bruker, Ettlingen Germany). A Bruker (Ettlingen, Germany) volume coil (inner diameter of 7 cm) and rectangular surface coil (1.7 × 1.4 cm) was used for signal transmission and reception respectively. The coil was tuned to the hydrogen proton frequency (300.3 MHz). The K_trans _values were obtained from serial images acquired to monitor changes in the concentration of a MR-visible contrast agent (GD-DTPA; Bayer Schering Pharma) within each pixel, during the initial uptake and subsequent washout of the agent in the tumor. The MRI scans follow the protocol reported by Lyng et al. [19]; briefly, mice were anaesthetized with isofluorane (5% induction, 2% maintenance), a catheter inserted into the lateral tail vein and the animal was placed supine with its head above the surface coil. A proton-density weighted scan was first acquired to serve as a baseline for conversion of pixel intensity to absolute concentration values of the contrast agent. A volume equivalent to 10 uL per gram body weight of the contrast agent (0.03 M Gd-DTPA in saline) was injected via the tail vein catheter in a period of 10-15 seconds. The contrast series consisted of a 3D RF-spoiled Fast Low Angle Shot (FLASH) sequence with timing and resolution parameters as follows: echo time/repetition time = 2.8/9.2 ms, Field of view = 1.92 × 1.92 × 1.6 cm, Matrix size = 128 × 128 × 16 cm, acquisition time per image = 9.45 seconds. Twenty baseline scans were acquired before contrast agent injection and 250 scans were acquired afterwards, resulting in a total acquisition time of 43 minutes. The concentration-time curve for each pixel was fit to a two-compartment Kety model [20] which describes the pharmacokinetics of the contrast agent using three parameters: ve (volume of extracellular extravascular space), K_trans _(volume transfer constant between the vasculature and tissue compartment) and Vp (fractional volume of the vascular compartment).

### *In vitro *endothelial cell exposure and nuclei count

For proliferative conditions, Dermal Human MicroVascular Endothelial Cells (d-HMVEC; 600 cells/well) and Human Brain Microvascular Endothelial Cells (HBMEC; 5000 cells/well) were plated in black 96-well plates (Optilux™, BD Biosciences, Mississauga, ON, Canada) and drugs were added the day after. For non-proliferative conditions, d-HMVEC cells (5000 cells/well) and HBMEC (50000 cells/well) were plated in black 96 well plates and drugs were added four days after. Irinotecan (Sandoz, QC, Canada), SN-38 (LKT Laboratories, MN, USA), vincristine (Novopharm, ON, Canada), docetaxel, paclitaxel (Taxol^®^; Bristol Myers Squibb Canada, QC, Canada) and doxorubicin (Adriamycin^TM/MC^, Pfizer, QC, Canada) were added in concentrations ranging from 1-100,000 picoMolar on cells and replaced daily for 7 days. At the end of drug treatment, cells were fixed with 3.5% paraformaldehyde (Electron Microscopy Sciences, PA) for 15 minutes at -20°C, permeabilized with 0.1% Triton (Perkin-Elmer, MA) in PBS for 10 minutes at room temperature, blocked for 1 hr at 4°C (Odyssey blocking buffer, Rockland, PA) and incubated overnight with Ki67 antibody (Invitrogen #18-0191z; 1:100 dilution in blocking buffer). Cells were then incubated with Anti-rabbit Alexa 488 secondary antibody (Molecular Probe #A11034, Invitrogen; 1:200 in blocking buffer) for 1 hr at room temperature. Nuclei were stained with Draq5 dye (Biostatus, Leicestershire, UK; 1:200 in PBS) for 30 min at 37°C. Twenty fluorescent photographs/well (Alexa 488 emission: 475 nm, excitation: 535 nm; Draq5 emission: 620 nm, excitation: 700 nm) were taken at 10 × magnification using an InCell Analyzer 1000 (Amersham Bioscience) and the total nuclei count (Draq5 stained nuclei) as well as Ki67 expressing nuclei count (Draq5 and Alexa 488 double stained nuclei) were quantified using InCell Developer Toolbox software (Amersham Bioscience, GE Healthcare, Baie d'Urfe, QC, Canada). Dose-response curves generated from total nuclei count were used to calculate drug concentrations causing a decrease in endothelial cell nuclei count by 20% (fraction affected: Fa = 0.2), 50% (Fa = 0.5), 75% (Fa = 0.75) and 90% (Fa = 0.9) and compared for both proliferative and non-proliferative cells. All data points represent the average of 3 independent experiments in triplicate +/- S.E.M.

### Statistical analysis

All statistical data was collected using GraphPad Prism (San Diego, CA). Because all treatment drugs were chosen based on previous rationale justifying their inclusion in the study, the experimental design should not be regarded as a screening assay and statistical analysis was done using the single comparison non-parametric two-tailed Mann Whitney test and no correction was made for multiple comparisons. All data are expressed +/- S.E.M.

## Results

### Irinophore C™, Caelyx^® ^and liposomal vincristine inhibit tumor growth and increase Hoechst 33342 delivery in subcutaneous GBM tumors

**Figure 1:**
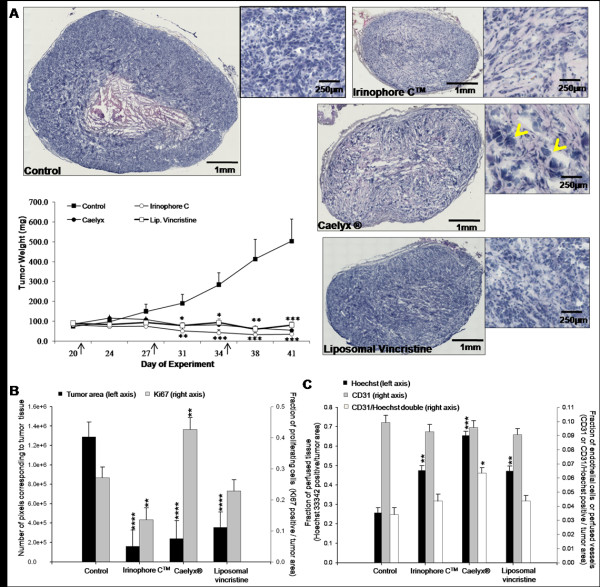
**Irinophore C™, liposomal vincristine and Caelyx^® ^significantly inhibits tumor growth, decreases proliferation and increases tumor perfusion in subcutaneous GBM tumors**. a) Representative H&E sections of tumors from each treatment group show the efficacy of the treatments in controlling tumor growth. Arrow heads indicate enlarged nuclei associated with Caelyx^® ^treatment. Tumor weights were calculated on the basis of caliper measurements; arrows indicate the treatment days. The Irinophore C™ statistical significance is indicated by bottom stars, while Caelyx^® ^and liposomal vincristine statistical significances are indicated by top stars (*p-value ≤ 0.05; **p-value ≤ 0.01; ***p-value ≤ 0.001) b) The area of viable tissue in tumor sections following treatment was expressed in number of pixels and correlates well with tumor volumes (■, left axis). The fraction of viable, actively proliferating cells (■, right axis) in the tumors was significantly decreased by Irinophore C™. Ki67 staining was also increased in Caelyx^®^-treated tumors. c) Hoechst 33342 perfusion in the tumors was increased significantly by Irinophore C™ and Caelyx^® ^treatment (■, left axis). The number of endothelial cells per unit area of viable tissue was unchanged by the treatments (■, right axis); however, the fraction of endothelial cells that were perfused (CD31 and Hoechst 33342 positive; □, right axis) was increased by treatment with Caelyx^®^. Statistical significances are indicated (*p-value ≤ 0.05; **p-value ≤ 0.01; ***p-value ≤ 0.001).

Rag2M mice bearing s.c. U251MG tumors (n = 9) were treated i.v. weekly for 3 weeks with 25 mg/kg Irinophore C™, 15 mg/kg Caelyx^® ^and 2 mg/kg liposomal vincristine. Tumor growth was monitored during the entire treatment period, and tumors were harvested 7 days after the last treatment. As noted in Figure 1a, the three drugs inhibited tumor growth significantly compared to untreated control (p < 0.05-0.001). At the end of the study (day 42), the weight of treated tumors ranged from 34 to 80 mg compared to an average of 502 mg for untreated control animals. A representative tumor section (H&E) derived from each treatment group is also provided in Figure 1a. The total non-necrotic tumor area (excluding necrotic and non-tumor area) measured in number of image pixels for each treated group is summarized in Figure 1b. The measurements of area of viable tumor tissue correlated with the tumor weight measurement and was significantly reduced for all treatment groups (compared to untreated tumors; p < 0.0001). The proliferation marker Ki67 was used to estimate the fraction of viable cells undergoing active proliferation within the tumor (positive Ki67 staining divided by total viable tissue, expressed as Ki67 positive fraction). Liposomal vincristine had no apparent effect on the Ki67 staining compared to control tumors. Treatment with Irinophore C™ caused a 2-fold decrease in Ki67 staining (p < 0.01). In contrast, a significant (p < 0.01) increase in Ki67 staining was observed in tumors from animals treated with Caelyx^® ^(Figure 1b). It should be noted that Caelyx^® ^treatment was also associated with enlarged tumor cell nuclei (see arrow heads in insert H&E image Figure 1a) and this may suggest that the treatment promoted cell cycle arrest [21]. This observation is in accordance with previously published findings on the effects of doxorubicin on cell cycle [22-24] and the fact that cellular Ki67 antigen has been shown to accumulate in some types of cell cycle arrest [25]. Finally, a decrease in number of cell nuclei per area (nuclei density) with a concomitant increase in connective tissue was observed by examination of the H&E stained sections in tumors from mice treated with Irinophore C™.

The effects of the selected liposomal drugs on tumor blood vessels were also evaluated. As summarized in Figure 1c, the CD31 staining (positive CD31 fraction) did not change significantly when comparing tumors from control animals to those from treated animals. Prior to sacrifice, animals were injected with Hoechst 33342, a marker for tumor perfusion that was previously validated by correlation with K_trans _measurements [8]. Total Hoechst 33342 staining in viable tissue (positive Hoechst 33342 fraction) was increased in the tumors obtained from treated animals (p < 0.01-0.001; Figure 1c). CD31 and Hoechst 33342 co-staining was measured to provide an indication of changes in functional blood vessels [8]. The results, summarized in Figure 1c, indicate that the number of functional blood vessels increased significantly (p < 0.05) in Caelyx^® ^treated tumors while there were no significant changes observed in tumors from Irinophore C™ and liposomal vincristine treated animals.

### Irinophore C™, Caelyx^® ^and liposomal vincristine inhibit tumor growth and decrease Hoechst33342 delivery in orthotopic GBM tumors

**Figure 2:**
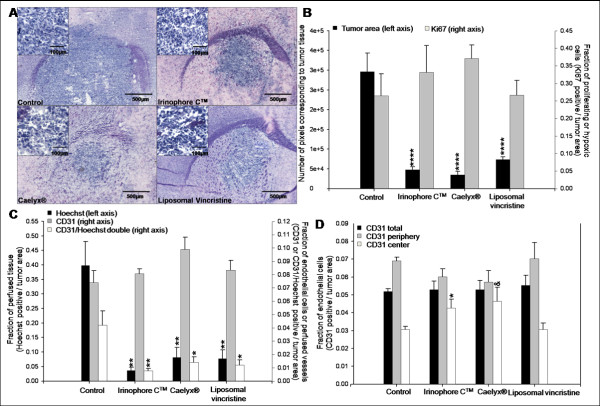
**Orthotopic GBM tumors treated with Irinophore C™, liposomal vincristine and Caelyx^® ^are significantly smaller than untreated controls**. a) Representative H&E images of brain sections from mice in each treatment group show that the area of tumor tissue (dark blue) from treated animals are smaller than untreated controls. b) Tumor areas were quantified in number of pixels, and used as a measure of treatment induced reduction of the tumor mass (■, left axis). No significant changes in proliferative activity (■, right axis) were observed. c) Hoechst 33342 staining was reduced significantly following treatments with the three liposomal treatments (■, left axis). The total number of endothelial cells per unit area of viable tissue (■, right axis) was unchanged across all groups, but the fraction of endothelial cells that were co-stained with Hoechst 33342 was significantly reduced (□, right axis). d) The density of endothelial cells (positive CD31 pixels divided by periphery or center tumor area pixels) in the center of tumors treated with Irinophore C™ was significantly higher compared to control tumors (□). No changes in endothelial cell density were seen in the total tumor area (■) or the periphery of tumors (■). Statistical significances are indicated (*p-value ≤ 0.05; **p-value ≤ 0.01; ***p-value ≤ 0.001). Non-significant trends are indicated (&p-value = 0.067).

Rag2M mice (n = 5 or 6) were inoculated with U251MG cells orthotopically (see Methods) and 21 days later the animals were treated i.v. (once weekly for 3 weeks) with 25 mg/kg Irinophore C™, 15 mg/kg Caelyx^® ^and 2 mg/kg liposomal vincristine. Forty-two days after cell inoculation, animals were sacrificed and their brains harvested. A representative tissue section (Hematoxylin and Eosin; H&E) showing the site of tumor growth (dark blue) within the brain of treated animals is provided for each treatment group in Figure 2a. Insert images have been included to show that following treatments, the tumor nuclear density drops slightly when compared to untreated controls. The average total non-necrotic tumor tissue in the tumor area for each treatment group was quantified to provide a measure of efficacy (Figure 2b). There was a significant reduction in tumor area for all treatment groups when compared to controls (p < 0.0001). In contrast to the results obtained with the s.c. glioma model, there was no significant changes in Ki67 staining observed following treatment (Figure 2b).

Prior to sacrifice, animals were also injected with Hoechst 33342. In tumors from untreated control mice, Hoechst 33342 staining was significantly greater in tumor tissue compared to matched regions of normal brain tissue (0.398 +/- 0.083 and 0.023 +/- 0.015 pixels/unit area, respectively; p < 0.01; data not shown). This staining pattern has been described elsewhere [26,27] and is consistent with the fact that Hoechst 33342 does not cross the blood-brain barrier. Interestingly, the data summarized in Figure 2c show that Hoechst 33342 staining in the orthotopic tumor tissue from animals treated with the liposomal drugs was significantly reduced (p < 0.01) when compared to tumors from control animals. The decrease in Hoechst 33342 staining in orthotopic tumors from treated animals was in marked contrast to treatment-induced increases in Hoechst 33342 staining noted for tumors derived from the same cell line (U251MG) and grown subcutaneously (Figure 1c).

No significant changes in overall CD31 staining (pixels/unit area) (Figure 2c) were noted in the orthotopic tumors obtained from treated animals (compared to controls). However, CD31/Hoechst 33342 co-staining was significantly reduced (p < 0.01-0.05) in tumors from treated animals when compared to control animals (Figure 2c). Moreover, treatment of orthotopic tumor bearing animals with Irinophore C™ was associated with a significant (p < 0.05) increase in CD31 staining in the center of tumors when compared to untreated tumors (Figure 2d; p < 0.05).

### Assessing vascular normalization in GBM tumors from animals treated with Irinophore C™, Caelyx^® ^or liposomal vincristine

**Figure 3:**
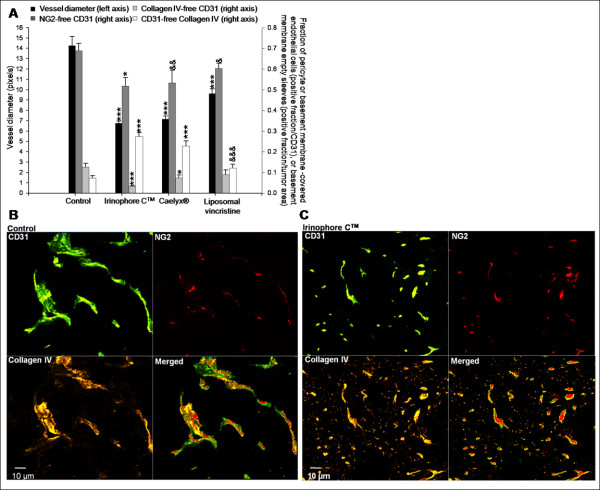
**Irinophore C™, liposomal vincristine and Caelyx^® ^treatments are associated with vascular normalization of the tumor vasculature in subcutaneous GBM tumors**. a) The diameters of tumor blood vessels were reduced significantly by all three treatments compared to control tumors (■, left axis). The fraction of NG2-free CD31 pixels (■, right axis), collagen IV-free CD31 pixels (■, right axis) were reduced in Irinophore C™ treated tumors indicating that fewer immature vessels are present following treatments. The proportion of empty basement membranes (CD31 free-collagen IV staining; □, right axis) in the viable tissue was also reduced by all liposomal treatments. b and c) Representative and merged images of CD31, Collagen IV and NG2 staining for control tumors (b) and tumors from Irinophore C™ treated mice (c). A reduction in blood vessel diameter (CD31; green) and an increase in basement membrane coverage of blood vessels (collagen IV; yellow) following treatment can be seen. Following Irinophore C™ treatment, more pericytes are present (NG2; red) and more endothelial cells are associated with the pericytes (merged image, green and red). Treatment with Irinophore C™ also results in an increase in empty basement membrane sleeves (i.e. CD31 free-collagen IV; yellow). The entire image represents non-necrotic and viable tissue. Statistical significances are indicated (*p-value ≤ 0.05; **p-value ≤ 0.01; ***p-value ≤ 0.001; ****p-value ≤ 0.0001). Non-significant trends are indicated (&p-value = 0.121; &&p-value = 0.071; &&&p-value = 0.054).

Several structural determinants, described as indicators of vascular normalization [28-30], were assessed in the orthotopic and s.c. GBM tumor models following treatment and these data were compared to tumors from untreated control animals. The parameters evaluated included: (i) the extent of discontinuous basement membrane (collagen IV-free CD31 pixels) in the tumor tissue, (ii) the fraction of pericyte-uncovered blood vessels (NG2-free CD31 pixels) in the tumor tissue and (iii) the blood vessel diameter. Furthermore, the proportion of empty basement membrane sleeves (CD31-free collagen IV pixels) was evaluated as an indication of regression of pre-existing blood vessels [9]. Treatment-induced changes in these factors are summarized in Figures 3 (s.c. tumors) and 4 (orthotopic tumors).

In s.c. GBM tumors, the fraction of NG2-free blood vessels was reduced by 25% in tumors from animals treated with Irinophore C™ (p < 0.05; Figure 3a). Decreases in NG2-free blood vessels were also noted in tumors from animals treated with Caelyx^® ^(p = 0.071) or liposomal vincristine (p = 0.121); but the effects were not considered significant. The number of collagen IV-free blood vessels was decreased in s.c. tumors from animals treated with Irinophore C™ or Caelyx^® ^(41-75% decrease; p < 0.05-0.001; Figure 3a). Blood vessel diameter was also reduced (32%-51%; p < 0.001) in s.c. tumors from all treatments groups. Finally, the number of empty basement membrane sleeves in tumors from Irinophore C™ and Caelyx^® ^treated animals was increased 3.4- to 3.8-fold following treatment (p < 0.0001). A similar effect was noted for tumors from animals treated with liposomal vincristine, but the effect was not considered significant (p = 0.054). Representative immunofluorescence micrographs highlighting the effects of Irinophore C™ treatment on the tumor vasculature of s.c. U251MG tumors (Figure 3c) compared to untreated tumor (Figure 3b) are provided to support the results summarized in Figure 3a.

**Figure 4:**
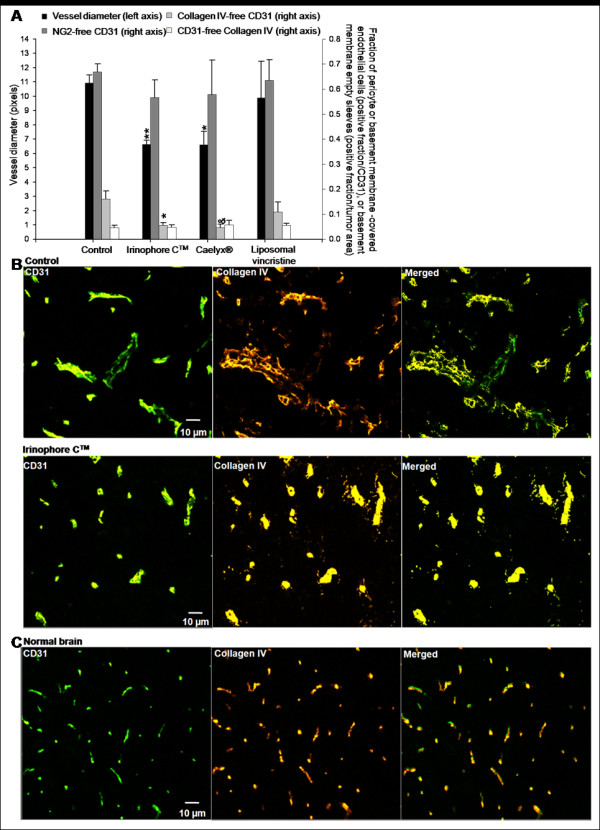
**Irinophore C™, liposomal vincristine and Caelyx^® ^treatments are associated with vascular normalization of the tumor vasculature in orthotopic GBM tumors**. a) Vessel diameters (■, left axis) and the fraction of collagen IV-free CD31 pixels (■, right axis) in orthotopic GBM tumors were reduced by Irinophore C™ and Caelyx^®^. However, no changes were seen in the fraction of NG2-free CD31 positive endothelial cells (■, right axis) or Collagen IV-free CD31 positive endothelial cells (□, right axis). b) Representative images from untreated and Irinophore C™ treated tumors; similar images for normal brain tissue are shown for comparison. (c) Blood vessel diameters (CD31; green) are reduced by Irinophore C™ treatment. The basement membrane (collagen IV; yellow) is partially restored by treatment with Irinophore C™. The entire image represents non-necrotic and viable tissue. Statistical significances are indicated (*p-value ≤ 0.05; **p-value ≤ 0.01). Non-significant trends are indicated (&p-value = 0.064).

Similar results were obtained when evaluating the orthotopic U251MG tumors from treated animals compared to controls. In addition, histological assessments of brain tissue surrounding the tumor allowed comparisons between vessels in the tumor vs. normal brain tissue. The fraction of collagen IV-free blood vessels in normal brain (0.049 +/- 0.015) was 69% lower (p < 0.05) than that observed in tumor tissue from control untreated animals (0.160 +/- 0.033), indicating that the organization of the basement membrane architecture is decreased in the tumor compared to normal tissue (data not shown). Tumors from animals treated with Irinophore C™ showed a significant 71% (p < 0.05) decrease in the fraction of collagen IV-free blood vessels when compared to tumors from control animals (Figure 4a). A similar effect was observed in tumors from animals treated with Caelyx^®^, but the effect was not considered significant (p = 0.064). In normal brain tissue, blood vessel diameters were 54% smaller (4.9 +/- 0.5 pixels; p < 0.0011) than blood vessel diameters observed in orthotopic tumor tissue obtained from untreated animals (10.9 +/- 0.6 pixels; data not shown). Orthotopic tumors from animals treated with Irinophore C™ or Caelyx^® ^exhibited a reduction in blood vessels diameters of 39% (p < 0.01; Figure 4a) when compared to control tumors. In contrast to results obtained with the s.c. tumors of treated animals, the level of empty basement membrane sleeves (Collagen IV-free CD31 staining) in the orthotopic tumors did not change following treatment (Figure 4a). It should be noted that the level of empty basement membrane sleeves in the normal brain tissue (0.035 +/- 0.009) was found to be similar to that measured in orthotopic tumor tissue from untreated animals (0.047 +/- 0.009) (data not shown). Treatments did not induce significant changes in fraction of NG2-free blood vessels (Figure 4a). The fraction of NG2-free vessels in the normal brain could not be evaluated as NG2 proteoglycan was found at the surface of polydendrocytes, a subpopulation of glial cells found in the brain [31]. Representative immunofluorescence micrographs illustrating the effects of Irinophore C™ treatment on the orthotopic tumor vasculature are provided in Figure 4b. Normal brain tissue sections are shown in Figure 4c for comparison.

### Magnetic resonance imaging-measured changes in vascular permeability/flow (K_trans_)

**Figure 5:**
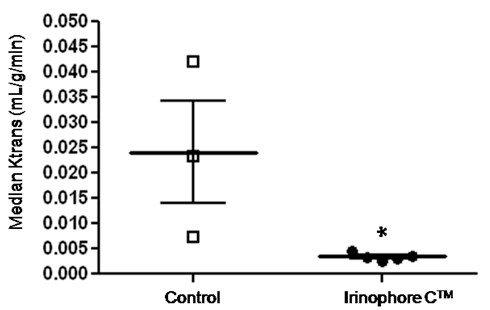
**Irinophore C™ reduced K_trans _measures compared to values obtained from control orthotopic tumors**. Individual and median (thick line) K_trans _values for untreated and Irinophore C™ treated orthotopic tumors with standard error of the mean (thin line). Statistical significance is indicated (*p-value ≤ 0.05).

The results summarized thus far are consistent with the idea that following treatment of animals bearing GBM tumors with lipid-based nanopharmaceutical formulations of vincristine, doxorubicin and irinotecan, there is a "normalization" of blood vessel structure. When considering these effects along with the antitumor activity, the greatest effects were observed following treatment with Irinophore C™. In order to confirm the idea of a Irinophore C™-induced vascular normalization, non-invasive magnetic resonance imaging was used to assess K_trans_, a volume transfer constant of a solute between the blood vessels and extra-cellular tissue compartment, in orthotopic tumors grown in untreated and Irinophore C™treated mice. The median values of K_trans _for the tumors within the control and treated groups have been summarized Figure 5. The results demonstrate that the median K_trans _value in untreated tumors was ~7 times greater than in treated tumors (0.0232 and 0.0034 ml/g/min, respectively, p < 0.05). It should be noted that the values for K_trans _in tumors from untreated animals were more variable when compared to the tumors from treated mice (s.e.m ± 0.010 and ± 0.0003, respectively).

### *In vitro *studies on endothelial cells mimicking the extended drug exposure achieved when using liposomal drug delivery formulations

**Figure 6:**
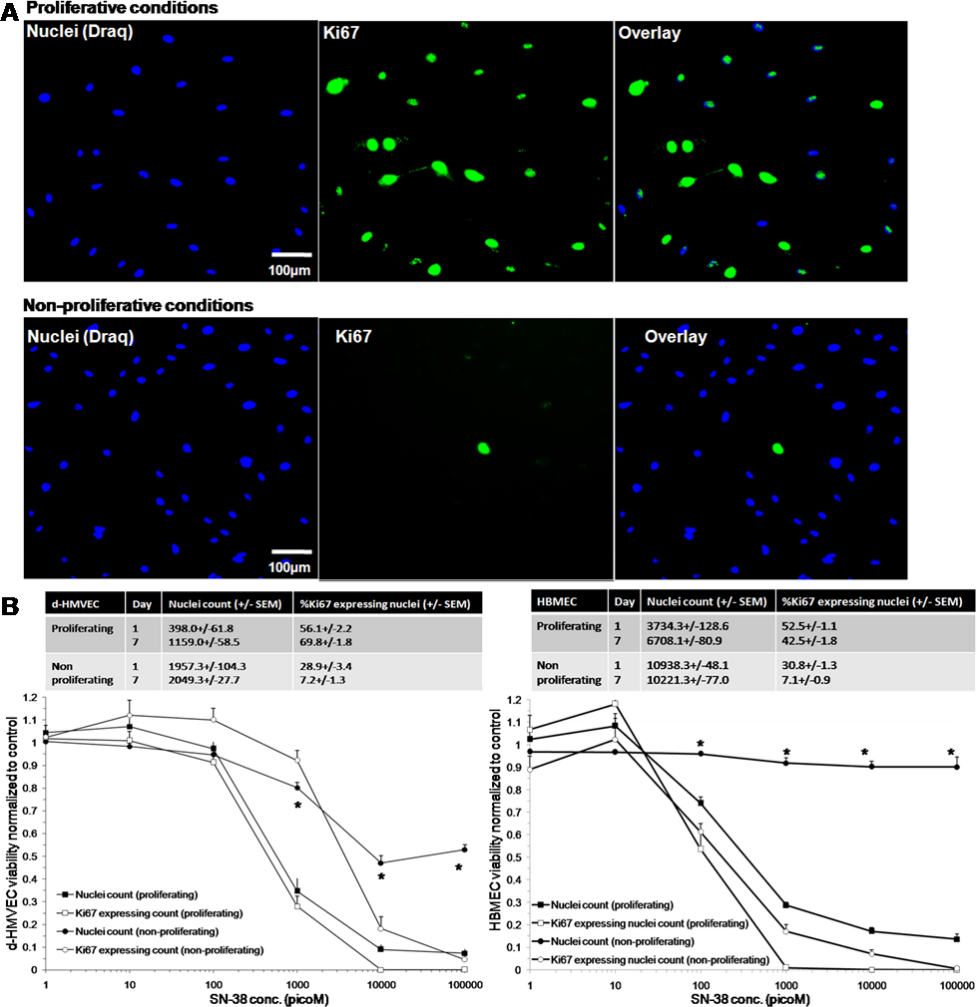
**d-HMVEC and HBMEC were plated under proliferative conditions or non-proliferative conditions**. a) Representative composite color images of d-HMVEC cells are shown; Draq (blue; nuclei), and Ki67 (green). Under proliferative conditions, the number of nuclei and Ki67 positive staining are similar; whereas under non-proliferative conditions, the number of nuclei with positive Ki67 staining is much lower. b) Total nuclei count and Ki67 expressing nuclei fraction of untreated cells for both cell lines under proliferative and non-proliferative conditions on day 1 and day 7 after plating (3 independent experiments; 3-21 replicates per experiment). Cells were exposed for 144hrs to increasing drug concentrations (1-100,000 picoM). Total nuclei count as well as nuclei expressing Ki67 expressing counts were normalized to counts obtained from control untreated cells. Representative data for d-HMVEC and HBMEC exposed to SN-38 is shown (3 independent experiments; 3 replicates per experiment +/- SEM). Statistical significance is indicated (*p-value < 0.0001) between total nuclei count of proliferative and non-proliferative cells.

In an attempt to better understand the effects of liposomal formulations used here on tumor vasculature, an *in vitro *endothelial cell assay was used to assess the impact of extended drug exposure. It is well established that these liposomal formulations engender significant increases in plasma drug concentrations over extended time periods following intravenous administration [7,11]. Thus, an extended drug exposure protocol was used to assess the effects of drugs in a model representative of the endothelial cells forming vessels in the subcutaneous or brain microenvironment. Dermal Human MicroVascular Endothelial Cells (d-HMVEC) and Human Brain Microvascular Endothelial Cells (HBMEC) were cultured under proliferative or non-proliferative conditions and exposed to the indicated drugs for 7 days. As illustrated in Figure 6a, the total nuclei count and the number of nuclei expressing the Ki67 proliferation marker were quantified using high content screening (Incell analyzer 1000) to discriminate between cytotoxic (reduction in total number of nuclei) and cell proliferation inhibitory effects (reduction in Ki67 expressing fraction). Under proliferative conditions, the nuclei count for the endothelial cell lines used increased up to 3-fold over the 7 day time period. The Ki67 expressing nuclei fraction ranged from 42 to 68% over this time frame (Figure 6a and 6b). Under non-proliferative conditions, the nuclei count for cell lines (d-HMVEC and HBMEC) remained unchanged from day 1 to day 7, and the Ki67 expressing nuclei fraction ranged from 7 to 31% (Figure 6a and 6b).

**Figure 7:**
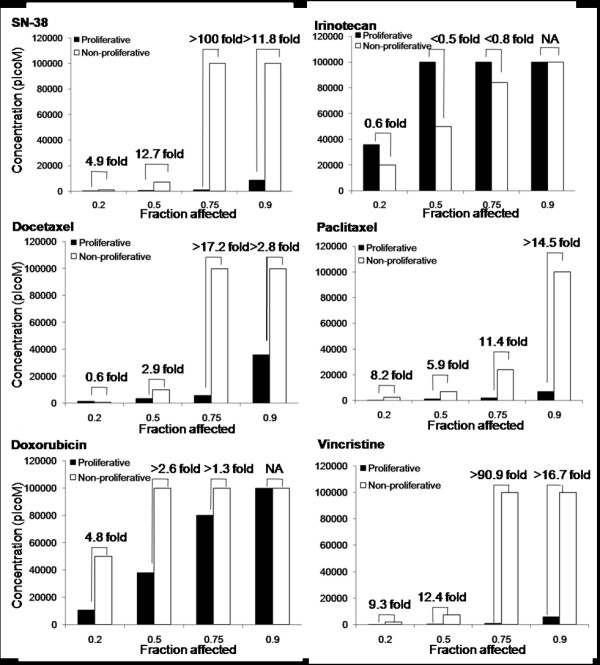
**Proliferating HMVEC cells are more sensitive to SN-38, docetaxel, paclitaxel, doxorubicin and vincristine than non-proliferating cells**. Concentrations at which a Fa of 0.2-0.9 was observed in d-HMVEC total nuclei count for both proliferative and non-proliferative conditions. The fold difference in drug concentration required to achieve the specified Fa is indicated above each pair of columns.

**Figure 8:**
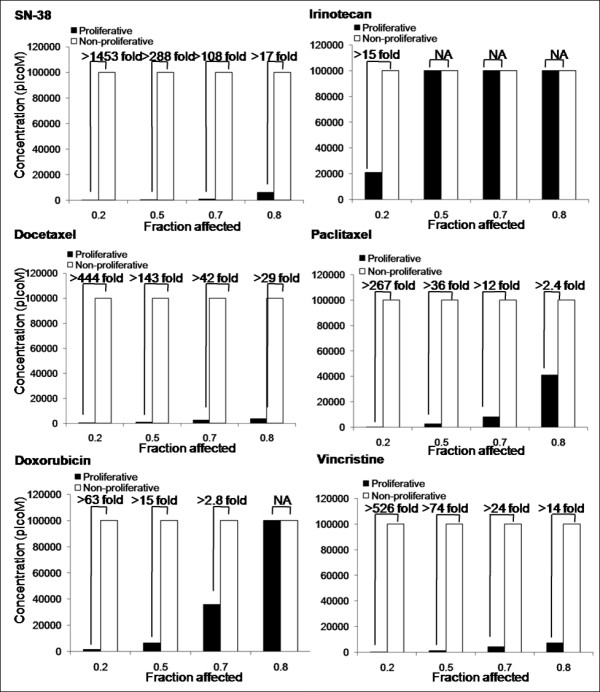
**Proliferating HBMEC cells are more sensitive to SN-38, docetaxel, paclitaxel, doxorubicin and vincristine than non-proliferating cells**. Concentrations at which a Fa of 0.2-0.9 was observed in HBMEC total nuclei count for both proliferative and non-proliferative conditions. The fold difference in drug concentration required to achieve the specified Fa is indicated above each pair of columns.

The activity of the drugs against the cells maintained under the two conditions was compared to assess the selectivity of the drugs for proliferating endothelial cells compared to non-proliferating endothelial cells. For all drugs used in this study, the dose-response curves for Ki67 expressing nuclei of proliferating cells matched the ones for the total nuclei count, suggesting that the drugs tested were cytotoxic rather than anti-proliferative. Representative dose-response curves for d-HMVECs and HBMECs exposed to SN-38, the active metabolite of irinotecan, under proliferative and non-proliferative conditions are shown in Figure 6b. The data indicates that SN-38 is significantly more active against proliferating endothelial cells then non-proliferating cells. In an effort to highlight differences in drug activity under proliferating and non-proliferating conditions, drug concentrations decreasing d-HMVEC total nuclei count by 20% (fraction affected: Fa = 0.2), 50% (Fa = 0.5), 75% (Fa = 0.75) and 90% (Fa = 0.9) were calculated from the dose-response curves and compared for both proliferative and non-proliferative cells (Figure 7). For example, results obtained at Fa = 0.75 indicate that the greatest differential effects were observed when using SN-38 and vincristine, where the drug dose required to achieve a 75% decrease in nuclei count under proliferation conditions were at least 100- and 90.9-times lower, respectively, than the drug dose required to achieve the same effect level under non-proliferative conditions. These effects were much greater than those seen using the positive control compounds docetaxel and paclitaxel. In contrast, there was little or no difference in the concentrations of irinotecan or doxorubicin required to achieve a Fa of 0.75 under proliferating and non-proliferating conditions. Similar results were obtained when using HBMECs (Figure 8). It should be noted that the drug doses required to achieve a Fa value of 0.5 for SN-38 and vincristine was 45 to 5000 times greater for U251MG glioblastoma cells when compared to the proliferating endothelial cells (data not shown) and the increased specificity for proliferating endothelial cells has been noted previously for paclitaxel and SN-38 when compared against human colorectal and breast cancer cells [32,33].

## Discussion

Studies over the last few decades have established that liposomal formulations of selected antineoplastic agents can be more effective than the same drug administered in free form. Liposomal formulations of anticancer drugs are known to have long circulation half-lives *in vivo*, and release the drug slowly over time [7,11]. Thus, the pharmacological properties of a drug given in its free form (e.g. via bolus injection or slow infusion) is changed dramatically by encapsulation in liposome. As a result, one might anticipate that the use of liposomal drugs will expose tumors to drugs for extended periods of time when compared to treatment with the free drug. This, of course, is well established in the literature and has been explained on the basis of the enhanced permeability and retention effect known to promote accumulation of intravenously administered liposomal drug formulations in tumors [34]. What is often not considered in studies with liposomal formulations is that these formulations constantly release the associated drug while in the circulation compartment, thereby extending the presence of the drug in the plasma compartment. This study tries to address whether part of the treatment benefits could be attributed to direct effects of the free drug (available in the blood compartment) on tumor vascular endothelial cells. The fact that these drug formulations are active against proliferating vasculature was anticipated, but not demonstrated to date. Liposomal drug formulations are known to accumulate and release drugs in close proximity to tumor blood vessels [14,15]. More intriguing, however, is the possibility that exposing the tumor vasculature to low concentrations of drug for extended periods may produce effects that are comparable to the vascular normalization effects described in the context of anti-angiogenic therapy [9,10] as discussed below.

In the present study, it is demonstrated that Irinophore C™, Caelyx^® ^and liposomal vincristine are effective against GBM grown subcutaneously or orthotopically (in the brain). The tumor masses in treated animals were significantly smaller compared to control (p < 0.001; Figure 1a), indicating that the liposomal drugs used in this study are potent against GBM, regardless of the site of tumor growth. Analysis of the tumor tissue, and in particular the vascular morphology, also indicates that treatments affected the tumor vasculature to various degrees. Overall, Irinophore C™ impacted the vasculature to a greater extent than the other formulations, and generated tumors with blood vessels that were morphologically more mature. In the subcutaneous model, Irinophore C™ restored the basement membrane architecture, increased the pericyte coverage and reduced blood vessel diameters. The data suggest a restoration of the vessel architecture to a more normal state. In the more clinically relevant orthotopic model, Irinophore C™ treatment restored the basement membrane architecture and reduced blood vessel diameters of the tumor vasculature, again suggesting a restoration of the vessel architecture to a more normal state. Irinophore C™ treatment also increased the quantity of vessel staining in the center of tumors, suggesting a more homogenous distribution of blood across the entire tumor. Further, Irinophore C™ reduced K_trans _values calculated from Dynamic Contrast Enhanced (DCE)-MRI studies significantly. Based on changes in vessel morphological appearance, the drop in K_trans _values was interpreted as a decrease in vessel permeability [35], and is consistent with the suggestion that Irinophore C™ treatment improved vascular function in the tumor. The larger variability in K_trans _values determined in tumors from control animals reflects the random nature of chaotic and leaky blood vessels in individual tumors [36]. It had already been established in s.c tumors that Hoechst 33342 could be used as a marker for tumor vessel function by validation with K_trans _measurements [8], but this had not been done for the orthotopic GBM tumor described here. It is shown here that the observed reduction in Hoechst 33342 staining after treatment while total CD31 staining remained constant correlates with a reduction in K_trans _measures. Taken together, these observations strongly suggest an improvement in vascular function. The tumor blood vessels in tumors from animals treated with Irinophore C™ behave more like vessels in the normal brain where the blood-brain barrier is intact.

The concept of 'blood vessel normalization' was first postulated in the 70s [37] and more recently, the clinical potential of vascular normalization has been described [9,10]. As with most solid tumors, the microvasculature of gliomas is characterized by tortuous and fenestrated vessels with diameters that are larger than normal [38] and discontinuous basement membrane which rarely encloses pericytes [39]. In glioma [28,29,40], antiangiogenic therapies can stop the growth of tumor vessels, prune immature and inefficient tumor vessels and normalize surviving vasculature by increasing the fraction of pericyte-covered vessels, restoring the abnormally thick and irregular basement membrane and reducing the high vascular permeability of these vessels [9,10]. In glioblastoma patients, a "vascular normalization index" was defined by changes in vascular permeability (K_trans _values), microvessel volume and circulating collagen IV. It was found that this index was closely associated with overall survival and progression-free survival in response to Cediranib, a pan-VEGFR inhibitor [40]. Pre-clinically, the delivery of temozolomide in an intracerebral model of glioma increased after treatment with the angiogenesis inhibitor SU5416. This drug restored capillary architecture and decrease interstitial fluid pressure [41]. Such studies offer strong evidence that the tumor vasculature in GBM is a valid target, and that therapies which 'normalize' tumor vasculature may improve the delivery of a second drug at some point in the treatment regimen.

The studies described here, together with an earlier publication [8], offer strong evidence that liposomal formulations of selected drugs, and especially Irinophore C™, induce a normalization of the tumor vasculature. In this study, collagen IV and NG2 were used as markers for basement membrane and pericytes, respectively. However, there is no consensus in the field for a definitive marker of these parameters. Other markers used to evaluate basement membranes include nidogen or laminin, and desmin or α-smooth muscle actin for pericytes [9,30]. These caveats notwithstanding, the morphological changes observed were associated with changes in Hoechst 33342 uptake in the tumor and when using this parameter, remarkably different results were obtained depending on the site of tumor growth (subcutaneous vs orthotopic). In the subcutaneous model, the liposomal treatments increased the amount of Hoechst 33342 staining in the tumor tissue (Figure 1c), while in the orthotopic tumors Hoechst 33342 staining was reduced (Figure 2c). As noted above, treatment effects were similar if blood vessel morphology parameters were used as a measured endpoint. While initially surprising, the Hoechst 33342 uptake data may actually be consistent with restoration of the blood-brain barrier, which is more impermeable to Hoechst 33342. It is well established that Hoechst 33342 is a p-glycoprotein substrate [42]. It does not accumulate in normal brain tissue because it cannot cross the blood brain barrier, but it is present in untreated orthotopic brain tumors which exhibit leakier blood vessel. This idea was further confirmed by K_trans _measurements, which strongly suggested a vasculature normalization induced by Irinophore C™. This interpretation suggests that Hoechst 33342 is not an appropriate marker for tumor *perfusion *in orthotopic glioma models, as it was previously used in a s.c. tumor model [8]. It does, however, function as a *permeability *marker for perfused tumor associated blood vessels, which is reduced upon normalization. The impact of vascular normalization on tumor perfusion in orthotopic GBM tumors could not be assessed in the present study because MRI K_trans _data and Hoechst 33342 staining data are not direct measures of perfusion in the brain tumor. However, data obtained in the subcutaneous model suggest that treatment with liposomal drugs does not reduce tumor perfusion, as measured by CD31/Hoechst 33342 double staining, and may even increase it, as suggested by data obtained from Caelyx^®^-treated s.c. tumors. Studies to measure the delivery of a second drug that can cross the BBB in liposomal drug-treated tumors are underway and will provide an indication of the impact of vascular normalization on vessel perfusion in the orthotopic model.

The idea that liposomal formulations of anti-cancer drugs, in addition to having a direct cytotoxic effect on the tumor cells, may also act as through anti-angiogenic mechanisms is intriguing. It seems reasonable to suggest that the extended drug release characteristics associated with the liposomal drug formulations used in this study [7,11] may have effects on blood vessels in a manner similar to metronomic dosing schedules - i.e. frequent, low dose administration of drugs with no prolonged drug-free breaks [43]. Metronomic dosing is now acknowledged to act specifically on the proliferating endothelial cells of tumor blood vessels [44] and was more recently shown to improve tumor perfusion and to decrease hypoxia in a pancreatic tumor model [45]. To examine this hypothesis, an *in vitro *assay was used to evaluate the activity of irinotecan, doxorubicin and vincristine (the drugs encapsulated by liposome examined in this study) against proliferating endothelial cells. The assay was adapted from one developed by Bocci et al. to examine the effects of metronomic drug exposure against endothelial cells [33]. Previous reports suggest that docetaxel and paclitaxel have potent activity against endothelial cells in an *in vitro *metronomic dosing regime [32,33,46], so these drugs were included in the assay as positive controls. The effects of SN-38 were also evaluated in the assay because SN-38 is a more active metabolite of irinotecan generated by tissue and plasma carboxylesterases *in vivo *[47,48]. Further it has already been established that following treatment with Irinophore C™, high levels of SN-38 are maintained in the plasma compartment for extended time periods [7]. SN-38 levels may play an important role in the anti-cancer activity of Irinophore C™.

The *in vitro *metronomic dosing assay presented in Figures 7 and 8 suggest that vincristine and SN-38, like the taxanes (docetaxel or paclitaxel), are highly active against proliferating endothelial cells (Figure 6a-b). In contrast, free irinotecan has little specificity for proliferating endothelial cells over non-proliferating cells *in vitro*. The data for free vincristine corroborate the effects on tumor vasculature seen with the liposomal form of the drug used here, while the results obtained with free irinotecan, which is not specific for proliferating endothelial cells, is actually contradictory. Irinophore C™ was the most active of the three liposomal formulations used. The results in Figure 7 and 8 would strongly suggest that the activity of Irinophore C™may be explained by the high plasma levels of SN-38 generated following administration of the formulation [7,16]. Thus it can be concluded from the studies presented here that the active metabolite of irinotecan, SN-38, may be the agent promoting vascular normalization in the models used here.

Interestingly, the *in vitro *assay suggests that doxorubicin should have little specificity on proliferating endothelial cells, yet i.v. administration of Caelyx^® ^resulted in effects on the tumor vasculature that were comparable to those seen following administration of Irinophore C™. The reasons for this are unclear at present but may be related to disruptions in the production of hypoxia-induced VEGF caused by doxorubicin [49]. Previous studies completed using the rat intracranial 9L tumor model treated with a formulation of doxorubicin comparable to that used here [15] showed the presence of vascular breakdown and hemorrhage 48 hours after treatment. In contrast, the results summarized here were obtained using tumors harvested one week after the final treatment; thus the data here may reflect late effects on tumor vasculature. Further, 9L is a gliosarcoma cell line which exhibits a slower doubling time (34.9 hrs [50]) than the U251MG glioblastoma cell line (20.9 hrs; data not shown) used in this study. The resulting 9L tumors are also histological distinct [50] when compared to the U251MG model. These differences will likely impact how tumors respond to agents capable of promoting vascular normalization. Studies assessing how vascular functions change in relationship to tumor growth rate are currently being completed.

## Conclusion

In aggregate, data from this study indicates that liposomal formulations of irinotecan, doxorubicin and vincristine exert anti-angiogenic effects, as measured by endpoints assessing increases in mature blood vessels and improved vascular function. The normalization of tumor vessels appears to be transient in nature [36] but may create a window where blood flow is improved, leading to an opportunity to improve drug delivery for other drugs. The fact that all three formulations were therapeutically active in the orthotopic model suggests that vascular normalization did not prevent the drugs from accessing tumor cells, despite the fact that our interpretation of data obtained from Hoechst 33342 suggests a reduction in vessel permeability. Data from our laboratory showed that once irinotecan is released from the lipid carrier, the drug and its active metabolite SN-38 are capable of crossing a normal blood-brain barrier (Verreault M, Strutt D, Masin D, Anantha M, Waterhouse D, Yapp DT and Bally MB: Irinophore C™, a lipid-based nanoparticulate formulation of irinotecan, is more effective than free irinotecan when used to treat an orthotopic glioblastoma model, submitted for publication in March 2011). Vincristine was also shown to be able to cross a normal blood-brain barrier [51]. Thus, it can be speculated that vascular normalization would increase the delivery of drug that have dissociated from the liposome across the tumor vasculature, allowing higher levels of drug to diffuse into a greater volume of tumor tissue. Studies assessing the consequences of liposomal drug-induced vascular normalization on the delivery of a second drug capable of crossing the blood-brain barrier will provide important information regarding the impact of tumor vessel permeability on drug delivery. In the case of GBM, an obvious choice of such a drug is temozolomide. Pre-clinical studies to assess the impact of Irinophore C™ treatments on the delivery of temozolomide are currently on-going.

## Abbreviations

d-HMVEC: adult dermal human microvascular endothelial cells; Fa: fraction affected; FBS: fetal bovine serum; GBM: glioblastoma; H&E: Hematoxylin and Eosin; HBMEC: Human brain microvascular endothelial cells; i.v.: intravenous; s.c.: subcutaneous;

## Competing interests

The authors declare that they have no competing interests.

## Authors' contributions

MV carried out all parts of the experimental manipulations, data analysis and draft of manuscript. DS and DM were involved in the implantation of s.c. and orthotopic tumors and monitoring of the animals. MA and DW were involved in the development of Irinophore C™ formulation. AY and PK were part of MRI-DCE data acquisition and analysis. MBB and DTY were involved in the conception of the study, participated in its design and helped to draft the manuscript. All authors read and approved the final manuscript.

## Pre-publication history

The pre-publication history for this paper can be accessed here:

http://www.biomedcentral.com/1471-2407/11/124/prepub
